# Patients’ perception towards digital health services in Saudi Arabia: A cross-sectional study

**DOI:** 10.1097/MD.0000000000036389

**Published:** 2023-12-15

**Authors:** Amal. H. Mohamed, Manar Ghonim, Mohammed Somaili, Amani Abdelmola, Ibrahim Yahya Ali Haqawi, Yasser Mohammed Nasser Shmakhi, Basmah Adel Ibrahim Refaei, Eman Adel Ibrahim Refaei, Amani Baker Aburasain, Maram Hafiz Ali Harbi, Reem Hafiz Ali Harbi, Osama Albasheer

**Affiliations:** a Department of Internal Medicine, Faculty of medicine, Jazan University, Jazan, Saudi Arabia; b Department of Community and Family Medicine, Faculty of medicine, Jazan University, Jazan, Saudi Arabia; c Medical Intern, Ministry of Health, Jazan, Saudi Arabia; d Medical Students, Faculty of medicine, Jazan University, Jazan, Saudi Arabia

**Keywords:** COVID-19, Healthcare Facilities, pandemic, perception, Telemedicine Network

## Abstract

The COVID-19 pandemic has highlighted the importance of the widespread use of digital health services (DHS). Despite evidence of the benefits of DHS, there are many barriers to their adaptation worldwide. This study aimed to measure the effectiveness of DHS from the patient perspective. A cross-sectional study was conducted in the Jazan region of Saudi Arabia from December 2022 to March 2023. Of the 323 participants who completed the online questionnaire, 63.5% were female, and 55.4% of participants found that DHS was satisfactory. 34% of the participants preferred DHS via telephone calls and 40.2% found that DHS was comparable to direct regular services in building trust between patients and doctors. A total of 79.2% agreed that DHS could reduce unnecessary outpatient visits and 70.9% agreed that it could be used effectively to follow patients with chronic diseases. DHS was found to be cost-effective in 76.8%. Digital healthcare has the potential to significantly improve health care outcomes and effectiveness in Saudi Arabia. Therefore, the use of a DHS for monitoring and dispensing care would be advantageous. However, difficulties such as lack of time or a packed schedule have prevented patients in Saudi Arabia from using telemedicine.

## 1. Introduction

**Figure 1.**
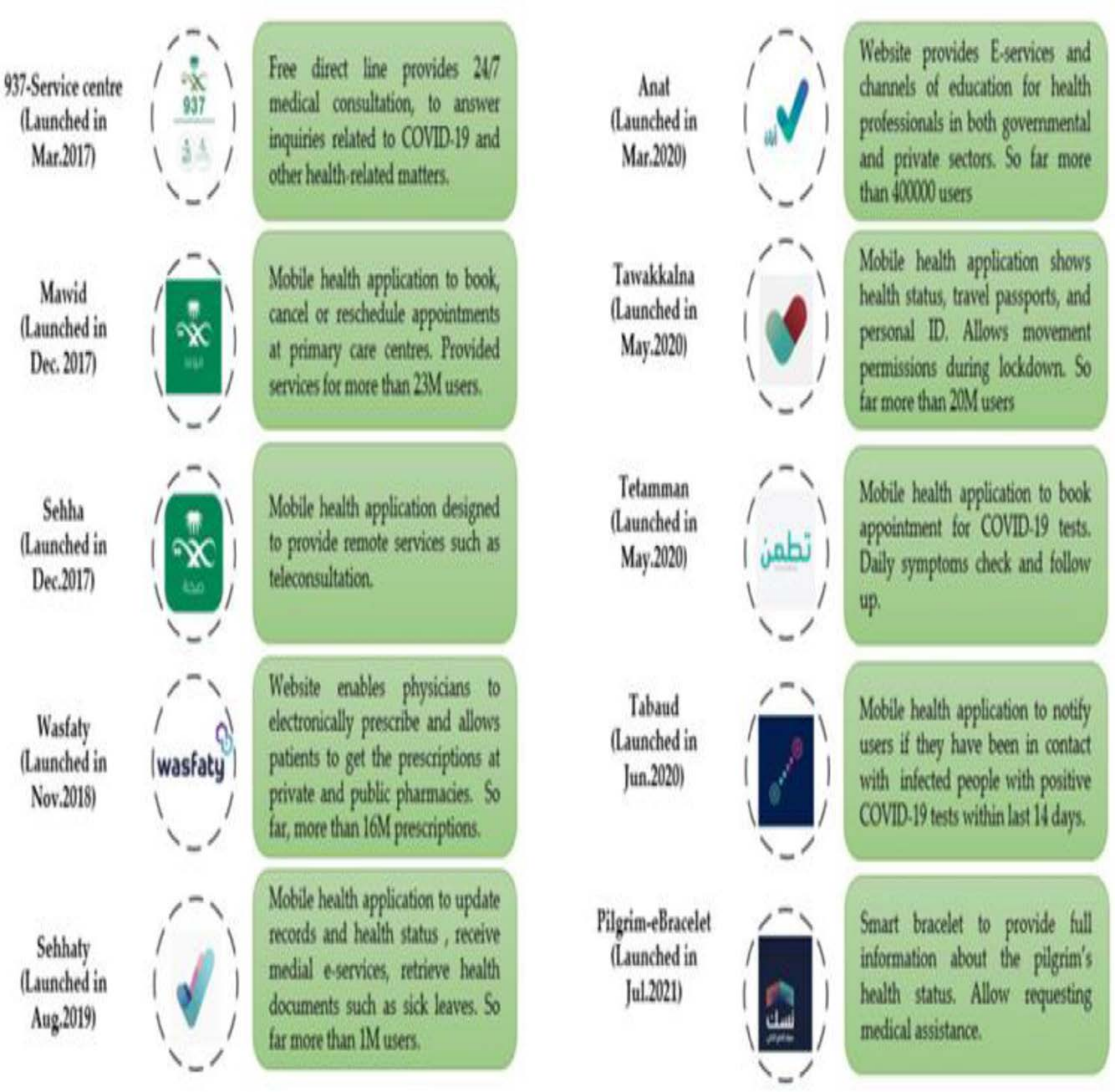
Different DHS Applications in Saudi Arabia. DHS = digital health services.

Digital health services (DHS) use e-health and communication networks to deliver healthcare services and medical education from 1 geographical location to another.^[1]^ In 2011, the Saudi Ministry of Health (MOH) launched the first national project for telemedicine, referred to as the Saudi Telemedicine Network (STN), covering all Healthcare Facilities (HCF).^[2]^ Despite the evidence regarding the great benefits and importance of DHS, there were many barriers to their adaptation, which was cited as a failed project (75%) and a percentage increase of up to 90% in developing countries.^[3,4]^ The COVID-19 pandemic and its restrictive measures have changed the health map not only in the KSA but also worldwide. This new situation represents a challenge in medical practice. Regular checkups, elective surgeries, and follow-ups are discouraged, and healthcare settings have become potential sources of COVID-19 infection.^[5,6]^ Healthcare workers faced waves of COVID-19 cases, significant shortfalls in personal protective equipment, a lack of social and institutional support, working extra hours, role conflict, ambiguity attributed to changing management protocols and incompetent training, and exposure to workplace violence.^[7,8]^ Saudi Vision (2030) has drawn up a roadmap to invest in digital healthcare in the coming decade. Telemedicine can be used for triage, direct care, follow-up, and consultation.^[9]^ The MOH in Saudi Arabia has recently introduced additional features to current TH services, which have been effectively incorporated into the provision of healthcare using mobile applications (e.g., Seha, Mawid, Tawakklna, Tabaud, and Tetamman) to cope with the pandemic.^[10]^ These health applications are illustrated in (Fig. 1).^[11]^

Several studies have been conducted on telemedicine and artificial intelligence (AI) to examine how Machine Learning (ML) algorithms and applications are employed in COVID-19.^[12,13]^ Sorkhabi et al outlined a methodological methodology for the preparation of electronic health records (EHRs), noting that it is necessary to extract more accurate and trustworthy knowledge.^[14]^

In addition to exploring the attitudes, experiences, and satisfaction of patients in the Jazan region, this study aimed to measure the effectiveness of DHS from the patient perspective.

## 2. Methodology

### 2.1. Study design and study area

This was a cross-sectional, questionnaire-based study. The first part of the questionnaire contained demographic characteristics of the participants, including age, sex, level of education, health status, and level of medical care. The second and third parts of the questionnaire included the participants’ perceptions and experiences of telemedicine on a 5-point scale that included the relationship between patients and physicians, assessment and accuracy of diagnoses, and their experiences. This study was conducted in Jazan, between December 2022 and March 2023. The Jazan region is located in the southwestern part of the Kingdom of Saudi Arabia, directly north of the border with Yemen, and along the east shore of the Red Sea.

### 2.2. Study population and sample size

The participants in this study were convenience samples and an electronic questionnaire was distributed through social media. All patients with chronic diseases were invited to participate in this study. The inclusion criteria were: age 18 to 60 years, residents in the Jazan area, reporting a diagnosis of chronic disease, having received a minimal consultation session through telemedicine, and willingness to participate in the study. Cochran equation $\left(x=Z{}^{2}pq÷e{}^{2}\right)$; where x = sample size, Z = Z score for CI, e = margin of error, and P = population proportion, was used to calculate the sample size with an acceptable reliability of α = 0.72. Using a margin error of 0.5% and 95% confidence interval (CI) with an estimated proportion of 0.5, and a population size of 1248427 (adult between 18 and 60 years) according to the 2018 survey, the estimated sample size was 385.

The data were tabulated and analyzed using descriptive and inferential statistics. After the analysis, the data were illustrated in tables and figures and expressed as frequencies, percentages, means, and medians with standard deviations. The chi-square test and regression model were used. The Statistical Package for Social Sciences (SPSS) version 28 was used for data management.

### 2.3. Ethical considerations

Participation in the study was voluntary, and the participants were free to participate and withdraw from the study at any time without any repercussions. This study was approved by the Standing Committee for Scientific Research, Jazan University (HAPO-10-Z-001), Reference No.: REC-44/04/345.

## 3. Results

With a sample size of 385 participants, 323 participants completed the survey, with a response rate of 83.9%. In terms of demographic characteristics, the gender distribution revealed that females were somewhat more likely than men to obtain virtual consultations (63.5% of women vs 36.5% of men). Forty to sixty years old (37.5%) was the age group that requested this service most frequently.

Regarding level of education, 85.5% were university or post-graduate, 14.2% were primary and secondary school, and only 0.3% were illiterate. Of these, 41.2% were employees and 39.9 were students. The questionnaire was distributed to all Jazan regions and patients of all nationalities: 60.1% of respondents from urban areas and 39.9% from rural regions (Table 1).

**Table 1:** Demographic characteristics of the participants (n = 323).

Variable	Values	Frequency	%
Age	18–29 yr	160	49.5
	30–39 yr	42	13.0
	40–60 yr	121	37.5
Gender	Female	205	63.5
	Male	118	36.5
Residence area	Urban	194	60.1
	Rural	129	39.9
Level of education	Illiterate	1	.3
	Primary and intermediate school	3	.9
	Secondary school	43	13.3
	University	238	73.7
	Postgraduate	38	11.8
Employment	Employee	133	41.2
	Student	129	39.9
	Unemployed	44	13.6
	Other	17	5.3

The 5 most common health statuses and diagnoses were hypertension (14.2%), diabetes mellitus (10.5%), gastrointestinal disease (10.5%), chronic respiratory disease (5.0%), and sickle-cell anemia (3.4%). In total, 55.4% sought digital health services for other diagnoses. The majority of the participants (57%) were diagnosed more than 1 year but <10 years ago, 34.1% were unsure about the exact time of their diagnosis, and 21.1% were diagnosed less than 1 year later. Regarding the level of medical care, 49.5% sought general hospital care, 30% sought primary health care, and 19.8% sought specialized centers. In 36.6% of cases, the health facility was not nearby (Table 2).

**Table 2:** Health status and level of medical care.

Variable	Values	Frequency	%
The diagnosed disease	Sickle cell anemia	11	3.4
	Diabetes mellitus	34	10.5
	Hypertension	46	14.2
	Chronic kidney disease	3	0.9
	Chronic respiratory disease	16	5.0
	Gastrointestinal disease	34	10.5
	other	179	55.4
Time since diagnosis	less than a year	68	21.1
	1–5 yr	57	17.6
	5–10 yr	40	12.4
	More than 10 yr	48	14.9
	not sure	110	34.1
Level of medical care provided	Primary health care unit	99	30.7
	General hospital	160	49.5
	Specialized center	64	19.8
Therapy compliance	Compliant	228	70.6
	Not compliant	95	29.4
Distance between home and health care provider	Nearby	205	63.5
	Far	99	30.7

**Figure 2.**
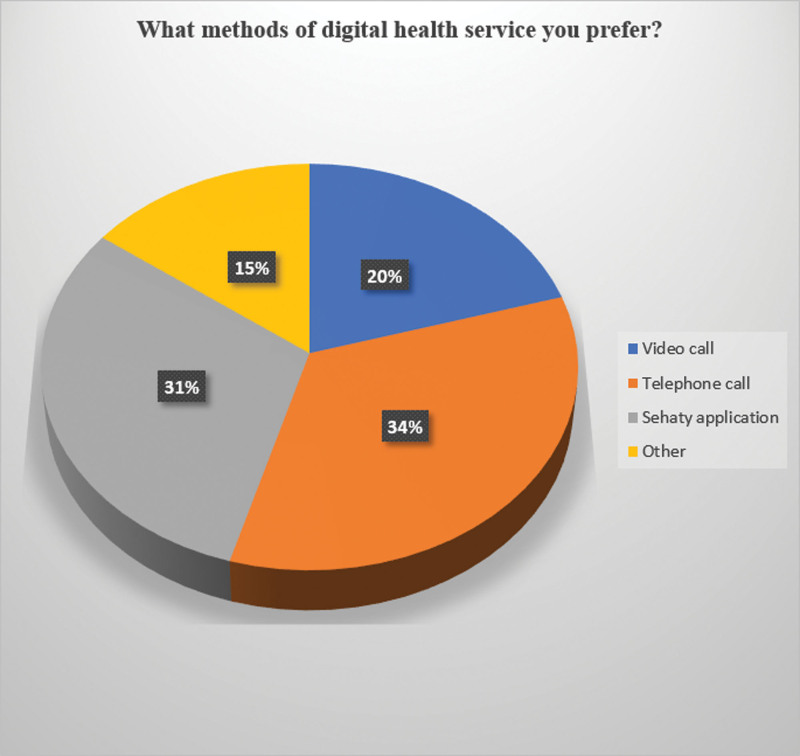
Preferred methods of digital health services.

34% Of the participants preferred Digital Health Services (DHS) via telephone calls, 31% preferred the Sehaty application, and 20% and 15% preferred video calls via other methods, respectively (Fig. 2).

The efficacy of DHS from patients’ perception covered 2 major domains: comparison between DHS and direct regular health services and participants’ experience with DHS.

### 3.1. Comparison between DHS and direct regular health services (Fig. 3)

40.2% found that DHS was the same as direct regular services in building trust between patients and doctors, while 35% felt it was better, and 24.8% felt the relationship on DHS was worse.

**Figure 3.**
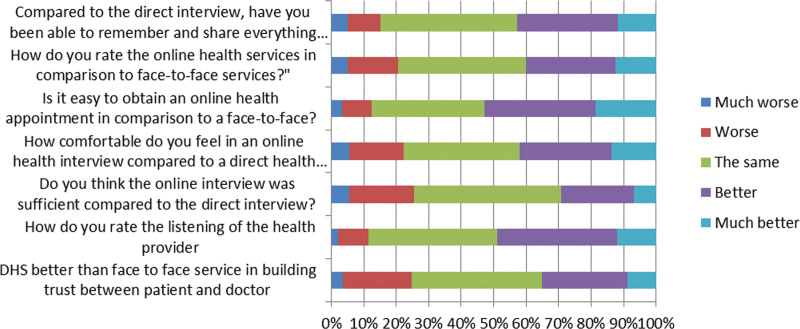
Comparison between DHS and direct regular health services. DHS = digital health services.

Of the participants, 36.8% thought that listening was better with telemedicine than with face-to-face listening, while only 9.3% thought it was worse. Approximately 45% of respondents found that online interviews were the same as direct interviews in terms of accuracy. 28.5% felt more comfortable in an online health interview, 35.6% thought there were no differences compared to a direct health interview, and 16.7% felt less comfortable in online interviews.

More than 50% found it easy to obtain an online health appointment compared to face-to-face appointments, 34.7% thought it was the same, and 12.4% reported that obtaining online appointments was not easy compared to direct services.

Compared with the direct interviews, 42.8% were able to remember and share everything about their health with the doctor, 15.2% found it worse to remember, and 42.1% thought it was the same.

In comparison to direct services, 27.6% and 12.4% rated online health services as better and much better, respectively, while 39.6% felt it was the same; 15.5% and 5.0% found it was worse and much worse, respectively.

### 3.2. Participants’ perception and experience towards telemedicine (Fig. 4)

A total of 79.2% agreed that telemedicine could reduce unnecessary outpatient visits (23.2% strongly agreed, 56.0% agreed), 13.6% were neutral, and only 7.1% disagreed.

**Figure 4.**
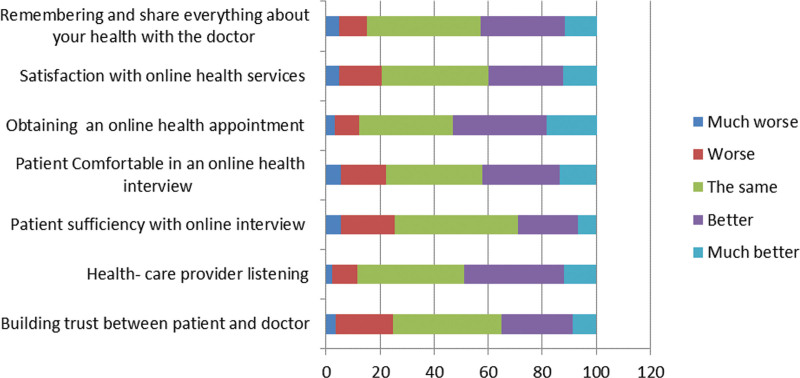
Participants’ perception and experience towards telemedicine.

69.9% thought that specialty affects efficacy of telemedicine service provided and 70.9% agree that it can be used effectively to follow patients with chronic diseases.

According to the participants’ experience, digital health services were an effective tool for providing patient care in 58.8% of participants, while 11.7% found it ineffective.

Regulations of digital health services practice were clear in 59.2%, and it was not unclear in 14%, and it is found to be cost-effective in 76.8%.

55.4% of participants found that services provided by digital health services are satisfactory, while 11.8% were not satisfied.

Linear regression was performed to evaluate the correlation between demographic characteristics, health status, medical care, and perception and experience of DHS. The demographic factors of perception and experience are presented in Table 3 & 4.

**Table 3:** Demographic factors with perception and experience (health status and level of medical care).

Coefficients^a^						
Model		Unstandardized coefficients		Standardized coefficients	t	Sig.
		B	Std. error	Beta		
1	(Constant)	15.710	1.743		9.015	.000
	Age	−.542	.207	−.151	−2.620	.009
	Gender	−.753	.398	−.109	−1.890	.060
	Residence area	.518	.377	.076	1.376	.170
	Education	−.187	.331	−.032	−.566	.572
	Employment	−.186	.224	−.048	−.828	.409

Predictors: (Constant), age, gender, residence area, education, and employment.

Dependent Variable: Participant perceptions and experience toward telemedicine.

The residence area factor has a highly significant positive relationship with the dependent variable, while the other factors have no such relation.

a Dependent variable: Health status and level of medical care.

* Demographic factors with perception and experience.

## 4. Discussion

The COVID-19 pandemic has caused a fundamental change in healthcare service delivery worldwide, with a dramatic increase in digital health services and virtual care.^[15]^

Telemedicine plays a crucial role in the COVID-19 pandemic in preventing morbidity and in avoiding high-risk sites of exposure in the general population. Additionally, elderly patients, who are at a high risk of developing more serious COVID-19 disease and its complications, might use electronic gadgets to access health services. Therefore, this study aimed to evaluate the efficacy of DHS during COVID-19 period from the patient perspective in the Jazan region. Patients are the main source of feedback on whether healthcare is being provided effectively and whether the care they receive satisfies their expectations. Therefore, patient satisfaction and perception are crucial for the effective implementation of healthcare services, and telemedicine is no exception.

In this study, 55.4% of participants were satisfied with healthcare provided by digital health services, which is consistent with a cross-sectional study conducted in Saudi Arabia by Thirunavukkarasu et al, who found a high satisfaction rate of (54.7%) and a virtual telemedicine clinic.^[16]^ Regarding the general caliber of the care provided and the overall telemedicine consultation experience, Nasser et al reported extreme satisfaction of 37.4% and 36.7%, respectively, expressed by the respondents, and 52% overall satisfaction with telemedicine.,^[17]^ whereas other studies from the UAE and the USA reported high satisfaction rates (85.9% and 69%, respectively).^[18,19]^

The degree of acceptability of and satisfaction with telemedicine services in Saudi Arabia may be positively impacted by several factors. These factors include the advanced government policy on digital technology, accessibility of qualified and well-trained medical professionals, and advanced Internet services, in addition to sociocultural and economic factors. This is in line with other studies conducted in the USA and Australia.^[20,21]^ However, in developing countries, the situation is different because of poverty, low literacy, and high cost of services.^[22]^

Residence area had a highly significant positive relationship with participants’ perceptions and experiences of telemedicine (*P* = 0. 002). Of the participants, 63.5% lived near health facilities. It was ironic to find an inverse relationship between the patient home distance and the medical center he visited. This can be attributed to living in towns or cities with readily available digital services and higher education levels. A study on the relationship between the distance traveled by patients and their willingness to utilize telemedicine demonstrated a statistically significant correlation in which an increase in the distance needed to travel to the clinic was associated with an increased acceptability of patients’ utilization of telephone consultations. This is not surprising, as one of the biggest advantages and driving forces of telemedicine has been to allow patients to seek care in remote locations, especially specialty care.^[23]^

Other factors such as age, sex, education, and employment had no such relationship. Older patients may have leaked their knowledge of the modern digital services. Studies from the UAE and India have revealed a significant relationship between increased age and overall satisfaction.^[18,24]^

Of the study participants, 55.4% had other diseases not related to chronic diseases of major systems, followed by those with hypertension and diabetes (14.2% and 10.5%, respectively). The results show that more than 70% of patients with chronic diseases prefer digital health services, as they have established a diagnosis and are frequently medically stable. A study designed by Steve S. Kong et al, concluded that if frequent monitoring could be effectively achieved and maintained using telemedicine, it may lead to overall improvements in patient outcomes and sustained long-term benefits.^[25]^ The study population who frequently visited general hospitals or primary healthcare units was more usable for digital health services. This may be explained by overcrowding and difficulties in gaining appointments in health centers.

This study revealed an overall positive effect of telemedicine on the relationship between patients and doctors. Two studies from India and Australia showed a positive impact of telemedicine, and they were pleased to avoid long-distance travel.^[26,27]^ A high percentage of respondents found no differences between telemedicine and direct health services in terms of building trust between physicians and patients and accuracy of the diagnosis. This is similar to the findings of Collins et al who reported no distinction between telemedicine and face-to-face consultation.^[27]^

Patients with good therapy compliance prefer digital health services because they are stable and have improved disease outcome. A similar study monitoring cancer patients with mobile health applications observed positive improvements in patient-reported outcomes among patients who utilized mobile applications on their smartphones compared with those who only utilized traditional clinic visits.^[28]^

70.6% of the patients were compliant with therapy received via telemedicine consultation; this high adherence to medication is in accordance with a systematic review of randomized controlled trials (86%) and cross-sectional study conducted in India (71%).^[26,29]^ Waller and Gilbody reported a compliance rate of 56%.^[30]^ In 2006, Skinner and Latchford stated that virtual therapy is a more popular and effective alternative to direct consultation, and that it can help clients and therapists build strong therapeutic bonds.^[12]^ This indicates that noncompliance could be due to personal reasons rather than technological issues.

The preferred method for telemedicine was telephone calls (34%) versus video calls (20%). This is similar to the results of the previous studies reported by Sloan et al and Al-Samarraie et al, who found that patients, especially females, might be uncomfortable with video consultations or sharing images for teleconsultations, which is attributed to cultural/religious concerns,^[13,31]^ but in contrast to other studies in which video calls were the preferred method. This can be explained by the cultural background of the participants in our study. Calls and electronic health records (EHR) may improve accessibility to patient time and treatment even without face-to-face consultation, according to a prior study conducted in the USA, and they can also assist healthcare professionals in making decisions.^[18]^

Digital technology has several advantages for healthcare systems, including its economic benefits. In 2020, the McKinsey Global Institute predicted that digitalization of healthcare would produce $250 billion to $420 billion in worldwide economic output by 2030.^[14]^ These cost savings can be reintroduced into other important health domains. One study reported 1000$ cost savings per child, and the majority of the patients (71%) had good experience with pediatric virtual consultation and cost savings.^[32]^ A study conducted in Sweden showed that a digital model of health services has a significant cost advantage on both the provider and patient side. The results show the financial and economic gross cost savings that can be realized if the digital care model is allowed to substitute the traditional care model at various rates of digital substitution.^[33]^

The majority of the participants stated that telemedicine is cost-effective, which is similar to several studies,^[26,32,34]^ which is in contrast to a previous study that found that medical technology leads to more expensive healthcare services.^[35–37]^

### 4.1. Limitation

A sample size of 323 was one of the limitations of this study because it cannot be regarded as wholly representative of Jazan entire population. Another limitation is that most participants had a high level of education, which may affect the generalizability of the results, as personal and demographic factors may play an essential role in participants’ attitudes and perceptions of DHS.^[38]^

## 5. Conclusion

In Saudi Arabia (KSA), digital healthcare has the potential to significantly enhance patient outcomes and efficiency.

The use of a DHS to monitor and provide care was considered beneficial. However, patients in Saudi Arabia are unable to use telemedicine because of issues such as lack of time or busy schedules. However, numerous persistent obstacles must be overcome to increase the awareness and rate of telemedicine use in several health professionals.

**Table 4:** Demographic factors with perception and experience (participant perceptions and experience toward telemedicine).

Coefficients^a^						
Model		Unstandardized Coefficients		Standardized Coefficients	t	Sig.
		B	Std. Error	Beta		
1	(Constant)	14.465	2.454		5.895	.000
	Age	.461	.291	.092	1.586	.114
	Gender	.061	.561	.006	.109	.914
	Residence area	1.617	.530	.170	3.049	.002
	Education	.070	.466	.008	.150	.881
	Employment	.232	.316	.043	.733	.464

Predictors: (Constant), age, gender, residence area, education, and employment.

Dependent Variable: Participant perceptions and experience toward telemedicine.

The residence area factor has a highly significant positive relationship with the dependent variable, while the other factors have no such relation.

a Dependent variable: Participant perceptions and experience toward telemedicine.

* Demographic factors with perception and experience.

## Acknowledgments

The authors are grateful to the participants of this study. Gratitude and thankfulness are extended to Dr Hisham Hanafi, PhD Business Administration, for his valuable assistance with data analysis. The authors also extend their appreciation to the Deanship of Scientific Research, Jazan University, for supporting this research through the Research Unit Support Program (Support Number: ISP23-47).

## Author contributions

**Conceptualization:** Amal. H. Mohamed, Manar Ghonim, Mohammed Somaili, Amani Abdelmola, Ibrahim Haqawi, Yasser Shmakhi, Basmah Refaei, Eman Refaei, Amani Aburasain, Maram Harbi, Reem Harbi, Osama Albasheer.

**Data curation:** Manar Ghonim, Mohammed Somaili, Amani Abdelmola, Ibrahim Haqawi, Basmah Refaei, Eman Refaei, Amani Aburasain, Maram Harbi, Reem Harbi.

**Formal analysis:** Mohammed Somaili, Amani Abdelmola, Yasser Shmakhi, Osama Albasheer.

**Funding acquisition:** Amal. H. Mohamed.

**Methodology:** Amal. H. Mohamed, Mohammed Somaili, Amani Abdelmola, Osama Albasheer.

**Project administration:** Amal. H. Mohamed, Manar Ghonim, Ibrahim Haqawi, Osama Albasheer.

**Resources:** Amal. H. Mohamed, Ibrahim Haqawi, Yasser Shmakhi, Basmah Refaei, Amani Aburasain, Maram Harbi, Reem Harbi.

**Software:** Amal. H. Mohamed, Yasser Shmakhi, Basmah Refaei, Eman Refaei, Amani Aburasain, Maram Harbi, Reem Harbi, Osama Albasheer.

**Supervision:** Amal. H. Mohamed, Manar Ghonim, Mohammed Somaili, Amani Abdelmola, Ibrahim Haqawi, Yasser Shmakhi, Basmah Refaei, Eman Refaei, Amani Aburasain, Maram Harbi, Reem Harbi, Osama Albasheer.

**Validation:** Amal. H. Mohamed, Manar Ghonim, Mohammed Somaili, Amani Abdelmola, Ibrahim Haqawi, Yasser Shmakhi, Basmah Refaei, Eman Refaei, Amani Aburasain, Maram Harbi, Reem Harbi, Osama Albasheer.

**Visualization:** Amal. H. Mohamed, Manar Ghonim, Mohammed Somaili, Amani Abdelmola, Ibrahim Haqawi, Yasser Shmakhi, Basmah Refaei, Eman Refaei, Amani Aburasain, Maram Harbi, Reem Harbi, Osama Albasheer.

**Writing – original draft:** Amal. H. Mohamed, Manar Ghonim, Mohammed Somaili, Amani Abdelmola, Ibrahim Haqawi, Yasser Shmakhi, Basmah Refaei, Eman Refaei, Amani Aburasain, Maram Harbi, Reem Harbi, Osama Albasheer.

**Writing – review & editing:** Amal. H. Mohamed, Manar Ghonim, Mohammed Somaili, Amani Abdelmola, Ibrahim Haqawi, Yasser Shmakhi, Basmah Refaei, Eman Refaei, Amani Aburasain, Maram Harbi, Reem Harbi, Osama Albasheer.
